# Psychophysical Determination of the Relevant Colours That Describe the Colour Palette of Paintings

**DOI:** 10.3390/jimaging7040072

**Published:** 2021-04-14

**Authors:** Juan Luis Nieves, Juan Ojeda, Luis Gómez-Robledo, Javier Romero

**Affiliations:** Department of Optics, Faculty of Science, University of Granada, 18071 Granada, Spain; jojojog@hotmail.com (J.O.); luisgrobledo@ugr.es (L.G.-R.); jromero@ugr.es (J.R.)

**Keywords:** colour, colour vision, paintings

## Abstract

In an early study, the so-called “relevant colour” in a painting was heuristically introduced as a term to describe the number of colours that would stand out for an observer when just glancing at a painting. The purpose of this study is to analyse how observers determine the relevant colours by describing observers’ subjective impressions of the most representative colours in paintings and to provide a psychophysical backing for a related computational model we proposed in a previous work. This subjective impression is elicited by an efficient and optimal processing of the most representative colour instances in painting images. Our results suggest an average number of 21 subjective colours. This number is in close agreement with the computational number of relevant colours previously obtained and allows a reliable segmentation of colour images using a small number of colours without introducing any colour categorization. In addition, our results are in good agreement with the directions of colour preferences derived from an independent component analysis. We show that independent component analysis of the painting images yields directions of colour preference aligned with the relevant colours of these images. Following on from this analysis, the results suggest that hue colour components are efficiently distributed throughout a discrete number of directions and could be relevant instances to a priori describe the most representative colours that make up the colour palette of paintings.

## 1. Introduction

The colourimetric analysis of pictorial works has aroused much interest in recent years, not only because of the study of the style used by the painter but also because of the computational applications that can be derived from the analysis of the statistical properties that characterize their spatial–chromatic content [1,2,3,4,5,6,7] and, additionally, the study of where a subject fixates their gaze when they observe works of art. There are several studies [8] that prove how eye fixations allow observers to perceive scene content and concentrate on local (i.e., lines, segments, shapes, or focus regions) and global (i.e., figure and ground, colour gamut, or edge distribution) features. Particular colour features were revealed as an important factor influencing visual attention during the visual search [9].

The question of how to pick out an appropriate colour palette from an image, and how to do that by reducing the number of distinct colours used in that palette, has been largely studied by many computer vision applications. In 1982, Heckbert [10] introduced an algorithm to display coloured images using only 256 colours, thus optimizing colour image quantization with the goal of a high-quality image display. We can find many examples of this issue, such as the public experiment offered by the Google Arts & Culture website [11], where you can find works of art that match your chosen colour palette, or the deep learning colour scheme generator by Colormind “to find good colours that work together in a colour palette” [12]. In practice, however, most of these approaches usually work with a fixed number of colours as a result of applying clustering and/or learning-based approaches. But, how is the human visual system (HVS) able to get a reliable (and reduced) colour palette from an image using no predefined colour categories? This is not a trivial question, and the answer is still a challenge for many computer vision applications. Different studies have characterized the properties of natural images with the aim of understanding our visual coding [13]. Pictorial works share essential statistical regularities with natural scenes, despite the fact that the dynamic range of the images is limited [14]. Amongst these properties, chromatic diversity has been introduced to analyse the colour gamut and colour volume expanded by natural and artificial images [15]. The estimation of chromatic diversity is quantified through the computation of the number of discernible colours in the images and has also aroused great attention because of the different applications and implications it has (e.g., gamut computation in displays, colour reproduction and rendering in museums, etc.). The general principle to estimate the number of discernible colours is to segment the colour space into just the noticeable sub-volumes and to count the number of these volumes containing colour representation of a minimum of one pixel. As it a classical problem with a long history, it can be stated that there are around 2 million distinguishable colours under daylight-type illuminant D65 (see [16] for a further review). However, the relationship between this number and the number of colours that really attracts the attention of an observer is still not clear and receives scant attention.

Nevertheless, colour naming and colour categorization have been extensively studied in recent years [17], not only because of the interest in knowing the mechanisms that the HVS uses to name colours using a limited colour lexicon, but also because of its direct applications in computational colour and object classification using machine-learning-related approaches [18,19].

In an earlier work we heuristically introduced the so-called “relevant colours” in a painting to describe the number of colours that would stand out for an observer when just glancing at a painting [20]. Our starting hypothesis in that study was that an observer would not be able to differentiate thousands of colours as determined when the usual discernible colour computations [5,15] are applied to either a natural or an artificial scene. Our proposed algorithm obtained an average number of 18 relevant colours, which means a huge percentage reduction in the number of colours in comparison with the initial thousand discernible colours found for each painting. The method derives different representative colours for each image and a segmented image linked to what we have interpreted as a representative colour palette that best matches the remarkable colour content of the image.

So far, scant attention has been paid to the influence of the observer’s task as far as the determination of the number of relevant colours describing the colour palette of a painting and/or the most remarkable colours in that painting is concerned. In this study, we developed a psychophysical experiment that aims to locate the relevant colours that describe the colour content of a painting without the need for a predefined colour categorization and/or colour naming paradigm. We studied whether the previous computation agrees with an experimental determination of relevant colours describing a painting, and which colour component, if any, would be guiding the subjective colour impression of the colours in the colour palette of paintings. Moreover, our results were analysed using an independent component analysis (ICA) to check whether the subjective impression is elicited by efficient and optimal processing of the most representative colour instances in painting images.

## 2. Materials and Methods

### 2.1. Image Datasets

**Figure 1:**
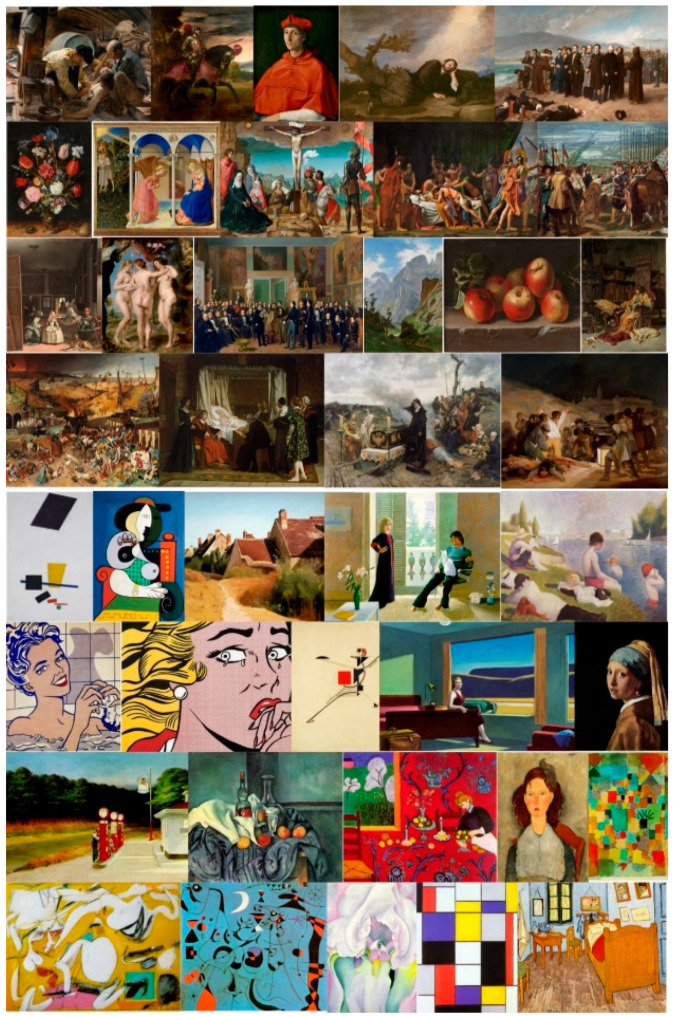
Paintings used in the experiment from (uppermost eight rows) the Prado museum [20] and from (lowermost eight rows) the Khan et al. dataset [22].

We collected 40 images (Figure 1) from two different sources: 20 paintings belonging to the Prado museum [21] and 20 images from the collection of the database of Khan et al. [22], which is publicly available on request. The former collection is a good selection of masterpieces exploring historical events as selected by the Prado museum for a 2-h visit; the latter collection includes the most relevant painters in Western art and covers painting styles from the Renaissance (15th and 16th centuries) to abstract expressionism (20th century). The images were used as they were included in the original database, which means that no additional post-processing was carried out.

The paintings of the Prado museum represent rural landscapes, flower vases, portraits, and indoor environments describing historical events. All the images from this dataset are characterized by complex spatio-chromatic content; the scenes represented in most of the paintings usually include many characters and a large variety of colours and objects at different distances. The width and height of the images varied from 1132 to 1920 pixels. The paintings in the second set are categorized both as abstract paintings and as easy indoor/outdoor scenes and do not include much complex spatio-chromatic content; in this set, the size of the images varied between 384 (width) and 1200 (height) pixels.

### 2.2. Procedure

The rationale of the computation of the number of relevant colours is as follows. A set of painting images was presented to different observers who had to find the set of colours that best describe each painting. The images were displayed using PsychToolbox3 [23]. In each trial of the experiment, the observer was instructed to select with the mouse an area within the image that contains a relevant colour, but what does a relevant colour mean? Observers visually scanned the whole painting and were asked to select those pixels belonging to a relevant chromatic area. It was not a colour categorization experiment and, thus, the observers were free to select as many colours as they liked. The observers sat 70 cm away from a self-calibrated model Eizo Color Edge CG277 27-inch monitor (EIZO Corporation, Hakusan, Japan) [24]; this monitor performs calibration periodically by the use of a built-in self-calibration sensor. Their head movements were restricted with a chinrest, and the presentation size of each image was adjusted to a maximum of 35 cm in width and 26.5 cm in height.

Each trial began with a fixation on a cross at the centre of the display for 10 s. Thus, each image was within the observers’ near peripheral field of view. Each observer continuously scanned the whole painting during the task; thus, they were using their 5° central or 8° para-central vision most of the time. The observers looked at a colour image and were asked to provide the number of relevant colours they perceived in the painting by clicking on representative locations within the painting that were considered valid instances of the relevant colours. The relevant colours were linked to remarkable colour areas that could be representative of the colour palette of the paintings according to the subjective impression of each observer. All forward computations were made by averaging 25 pixels around each pixel selection. The observers were free to select equally or similarly relevant colours from different spatial locations in the painting. It is known that 10 s is sufficient to obtain an overview of a picture, whilst 30 s is the average observation duration for an aesthetic judgment when unlimited time is given [25]. Thus, there was no time limitation per painting to finish the task, although no more than 60 s per image was recommended to avoid visual fatigue.

### 2.3. Observers

Six observers with normal colour vision (aged from 30 to 60, three women and three men) participated in the experiment. Two of them were naïve and were not familiar with the purpose of this kind of experiment. It is plausible to think that results could be different for art historians and art appraisal experts. It is very likely that their knowledge about the painting style, aesthetic, and/or painter biography would influence the choice of relevant colours. Thus, we preferred to exclude experts in art to avoid potential bias in the results at this stage.

The ethical research committee of the University of Granada provided ethical permission for the study.

### 2.4. Statistical Descriptors of the Paintings

The statistical descriptors used in this study were the colour gamut and distribution of CIEL*a*b* colour components; the limits, shape, and orientation of the colour gamut; the number of discernible colours; and four metrics to quantify the complexity of the image.

The number of discernible colours was computed by segmenting the CIEa*b* representation of each image into unit squares and counting the number of non-blank squares. As proposed by Linhares et al. [15], it was assumed that all the colours that were located within the same square, i.e., sharing a colour difference less than or equal to 1 CIEa*b*, would not be discernible. The colour volume of each image was defined as the palette of all the available colours in that image. The colour gamut was obtained by projecting the colour volume into the plane (a*, b*) of the CIELAB colour space. The limits, shape, and orientation of the gamut for each image were characterized by the properties of an ellipse fitted to the data based on a least square criterion. The area, axis ratio, and angular position of the longer axis of the fitted ellipses (with respect to the positive a* component) were also estimated for each colour gamut.

In order to characterize the image complexity, the following four image complexity metrics [26] were calculated from the RGB images used in the experiments:

- Self-similarity: by using the histogram intersection kernel and comparing the histogram of oriented gradients (HOG) features of each sub-image at level 3 with those of the entire image at level 0;
- Complexity: computed as the mean norm of the gradient across all orientations over the gradient image;
- Birkhoff-like metric: computed as the ratio between the self-similarity and complexity metrics;
- Anisotropy: calculated as the variance of all the HOG values at level 3.

In addition, an independent component analysis (ICA) was also applied to obtain the feature vectors that were adapted to the properties of the paintings. ICA allows the separation of the multivariate image pixel data into additive subcomponents. To do so, the FastICA 2.5 Matlab package (for Matlab 7.x and Matlab 6.x versions) [27] was used.

## 3. Results

**Figure 2:**
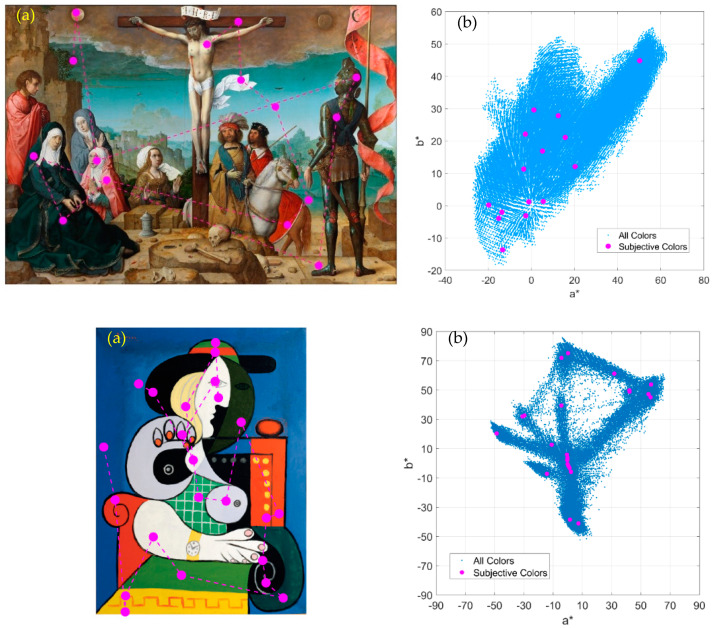
(**a**) Example of the colours selected as relevant colours (magenta circles) for one image from the Prado museum (*The Crucifixion*, by Juan de Flandes, 1509–1519) and one example from the Khan database (*Seated woman with wrist watch*, by Picasso, 1932). (**b**) CIEa*b* colour coordinates of all the colours within the image and the relevant colours chosen by one observer according to the subjective impression of the corresponding painting.

Figure 2 shows examples of the colours selected as relevant colours by one of the observers. Each magenta circle indicates all the colours that best describe the colour content of the image according to the subjective impression of that observer. Some colours seem to be quite similar but the spatial locations are different (e.g., the woman and man wearing red capes in the painting The Crucifixion). The right side of Figure 2 also shows the plots of all the CIEa*b* colours in the painting versus the a*b* colour components of the selected relevant colours for one of the observers. The colour gamut expanded by all the colours in the painting (illustrated by the blue points in the figure) is greater than the colour gamut expanded by the subjective colours selected (magenta circles in the figure). Nevertheless, the 15 experimental colours selected in this painting are spread around almost all the main areas of the original colour gamut of the image. This suggests that the observer was able to sample almost all the remarkable colour areas that describe the colour content of the image. This tendency and ability of observers is more clearly illustrated in the example chosen from the Khan dataset. The colour distribution shown in the lower right plot of Figure 2 shows a star-like shape of a* and b* colour components that is representative of the finite number of discrete colour areas which make up the original painting. That colour distribution was sampled from the 24 relevant colours selected by the observer. Similar results were obtained for all observers, which means that the observers were able to subjectively select a reduced number of colours that were representative of the remarkable colours describing the painting (amongst the potential thousand discernible colours that could appear in the image).

**Figure 3:**
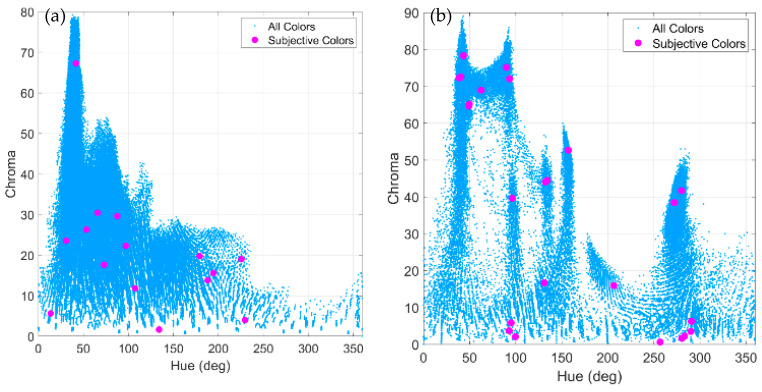
Distribution of hue versus chroma values for all the original colours and the subjective relevant colours selected by one observer for (**a**) *The Crucifixion*, and (**b**) *Seated woman with wrist watch*.

Figure 3 shows how the chroma and hue values influenced the observer’s selection of subjective relevant colours. The example shown on the right clearly illustrates how some paintings are usually composed of a number of remarkable hues as a function of the chroma values (clusters of red dots vertically aligned in the figure). In general, the observers were always able to select the relevant colours around the main peaked distributions of the colours in that chroma versus the hue representations, suggesting that the subjective impression of the observers was guided through instances of relevant hues appearing in the images.

**Figure 4:**
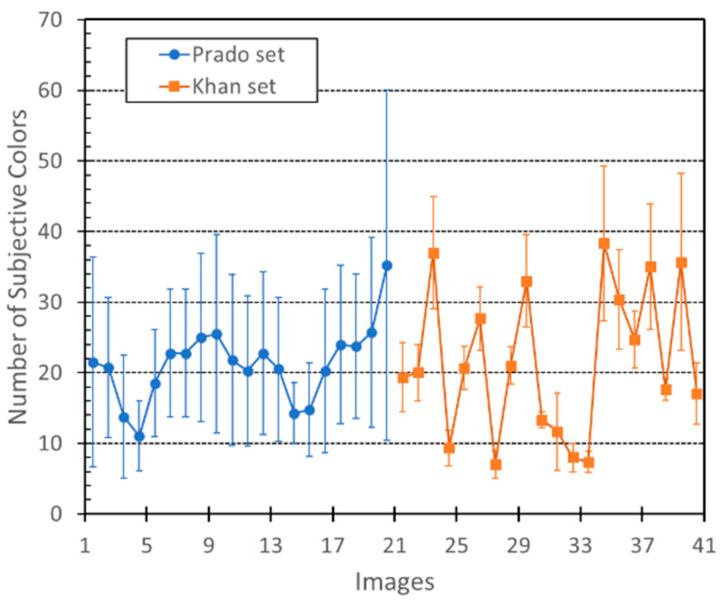
Average numbers of subjective relevant colours obtained for all painting images and all observers.

Figure 4 describes the average numbers of subjective relevant colours found by all the observers for the two image datasets used. The results show that there was almost no difference between the numbers of subjective colours obtained for the Prado museum set (21 colours with standard deviation of 5) and the Khan set (22 colours with standard deviation of 11). Nevertheless, the dispersion for the Khan dataset was greater than the corresponding value for the Prado museum paintings.

### 3.1. Subjective Relevant Colours versus Computational Relevant Colours

We applied our early computational approach in Nieves et al. (2020) [20] to estimate the relevant colours describing the colour palettes of both painting datasets. The number of subjective relevant colours found in the current psychophysical experiment (i.e., 21 colours) is in close agreement with the average number of 19 relevant colours (with standard deviation of 6) obtained computationally for the two datasets.

**Figure 5:**
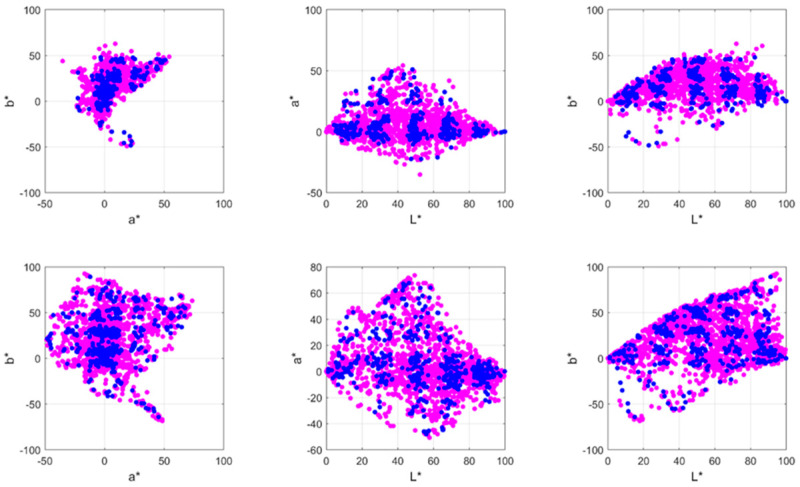
All computational (blue points) and subjective (magenta circles) relevant colours obtained from all observers. The upper row corresponds to the Prado museum paintings, and the lower row corresponds to the Khan image dataset.

**Figure 6:**
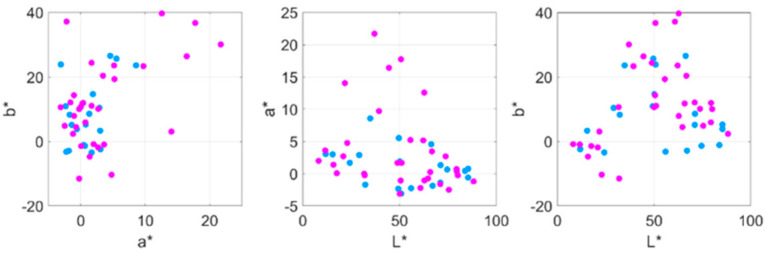
Comparison between the computational colours and relevant colours obtained for the painting *Queen Joanna the Mad*, by Pradilla, 1877 (which corresponds to image number 19, fourth row, in Figure 1) and one of the observers. Computational colours are marked as blue points, and the obtained experimental relevant colours are marked as magenta circles.

As is shown in Figure 5, there was good agreement between the CIEL*a*b* set of colours selected in the psychophysical experiment and the automatic relevant colours selected by the computational algorithm (i.e., both colour gamuts are similar). Although exact overlapping between the two sets of colours was not found, most of the computed relevant colours had their subjective colour counterparts (full data about the percentage of overlap between the computational and the experimental gamuts can be found in the Figure S1 in the Supplementary Materials section). Figure 6 shows an example of the results predicted by the algorithm and the subjective colours selected by one observer. Our results suggest that depending on the painting, the constraints imposed by the early algorithm proposed in Nieves et al. (2020) [20] are sometimes too restrictive. In addition, salient areas may influence the observer’s choice and should be considered [28,29]. Most of the subjective colours are close to the computational relevant colours, although some reddish colours (which are characterized by large a* values) were selected by the observers but not by the algorithm.

Table 1 shows the main colourimetric statistical and complexity descriptors estimated for the two sets of images. Both sets of selected images share similar chromatic properties, with slight differences for the chromatic axes with relatively small mean L* for the Prado set in comparison with the other image set. Other authors [7] have found statistical characteristics common to different painters. Most differences amongst artists can be found in the volume and area of the gamut, with the number of discernible colours being a parameter of large variability amongst painters. Although we have not analysed our results in terms of the painters (due to the reduced number of artists represented in our set of paintings) the volume results here also differ appreciably between the image datasets.

The complexity of the scenes was slightly different for the two image sets, although it depended on the metric analysed. However, there was no correlation between any of the aesthetic metrics used and the number of subjective colours found in the experiment (see Figure S2 in the Supplementary Materials). All the images maintained similar self-similarity, meaning that the paintings were exactly or approximately similar to a part of themselves. Nevertheless, the complexity of the Khan images was greater than the corresponding value obtained for the Prado paintings. Surprisingly, visual inspection of the images (as shown in the thumbnail in Figure 1) may lead to a contrary impression, but complexity is related to the gradients across all orientations over the gradient image and is not directly connected with the subjective impression of the spatio-chromatic image content itself. Anisotropy for the Prado set is clearly smaller than the corresponding value for the Khan set, indicating that the variance in the histogram of gradients was greater in the latter case. The Birkhoff metric was also similar between both kinds of images. As the Birkhoff metric is an aesthetic measure defined as the ratio between order and complexity, all the images selected in this study share more or less the same aesthetic subjective impression.

Our results also suggest that there is almost no difference between the numbers of subjective relevant colours obtained for the Prado museum images (21 colours with standard deviation of ±5) and the Khan images (22 ± 11 colours). These numbers are also in close agreement with the computational number of relevant colours if we apply the previous algorithm to these paintings (19 ± 6 relevant colours).

### 3.2. Colour Palette of Paintings Estimated from the Subjective Relevant Colours

If the observers selected the most representative image colours during the experiment, i.e., the colour palette of the painting, it is plausible that those colours could be appropriate seeds to segment the painting. If so, the segmented image would be quite similar to the original one or at least to the subjective impression obtained by the observer. Thus, from each set of subjective colours selected by the observers, we extracted the corresponding colours which make up the palette for the painting. This colour palette allows us to assign the relevant colour in the areas occupied by all the pixels close to that relevant colour. To do so, we built a look-up table (LUT) for each image using the corresponding set of relevant colours for that image. We computed the distance between every pixel colour in the original image and each colour in the LUT; by finding the minimum distance, each original colour was then transferred to a new relevant pixel colour.

**Figure 7:**
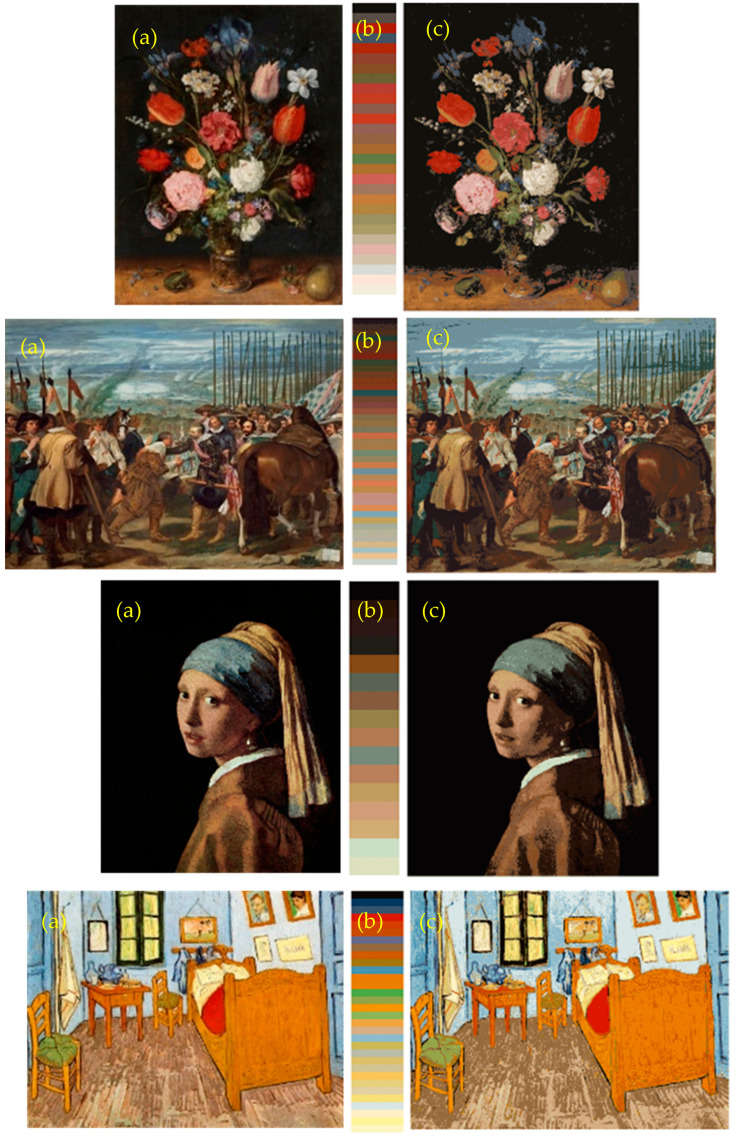
(**a**) Original painting and (**c**) its corresponding segmented counterpart based on the subjective relevant colours describing the colour palette of the painting (**b**). The numbers of relevant colours in each palette were 29, 41, 16, and 32, respectively, from the top to bottom images. The correlation coefficients between each original image and each segmented image were 0.9554, 0.9718, 0.9148, and 0.9249, respectively.

Figure 7 shows examples of the colour segmented images based on the relevant colours describing the psychophysical colour palette of the paintings. We computed the correlation between each RGB colour plane of the original and the corresponding RGB colour plane of the segmented image. The average correlation values for all the observers were 0.9408 (with standard deviation of 0.0059) for the Prado museum image dataset and 0.9552 (with standard deviation of 0.0017) for the Khan image dataset. Although these values suggest a non-perfect image recovery, the segmented images maintain the most remarkable colour content of the painting.

In our previous study [20], we managed a similar colourimetric segmentation of the image in question “as far as the discernible categorical colours which appeared in the image were concerned”. Nevertheless, those results were based on a computational relevant colour counting procedure (i.e., without any psychophysical basis), and thus, the image segmentations were only introduced as a potential application of the algorithm. We can now argue that the categorical colours included in the colour palettes shown in Figure 7 have subjective counterparts and are good colour instances to represent the subjective colour palette of paintings.

### 3.3. Subjective Relevant Colours, Colour Features, and Efficient Coding

Several previous studies have shown that the HVS efficiently encodes the colour information contained in natural scenes. According to the efficient coding principle proposed by Barlow [29], this means that the colour signal entering the eye, an inefficient representation of the natural raw input, is transformed by the HVS into an optimal output—an efficient representation of the input signal—with minimum loss (i.e., maximum information transmitted). ICA has been frequently used to find a linear transformation that allows the optimal output to be as statistically independent as possible [30,31]. By applying ICA to natural images, Watchler et al. [32] and, later on, Kellner and Watchler [33] showed how opponent coding of visual information is efficient for encoding colour inputs from natural scenes. These authors found different directions of colour preferences in addition to the classical red-green and blue-yellow opponent modulations.

**Figure 8:**
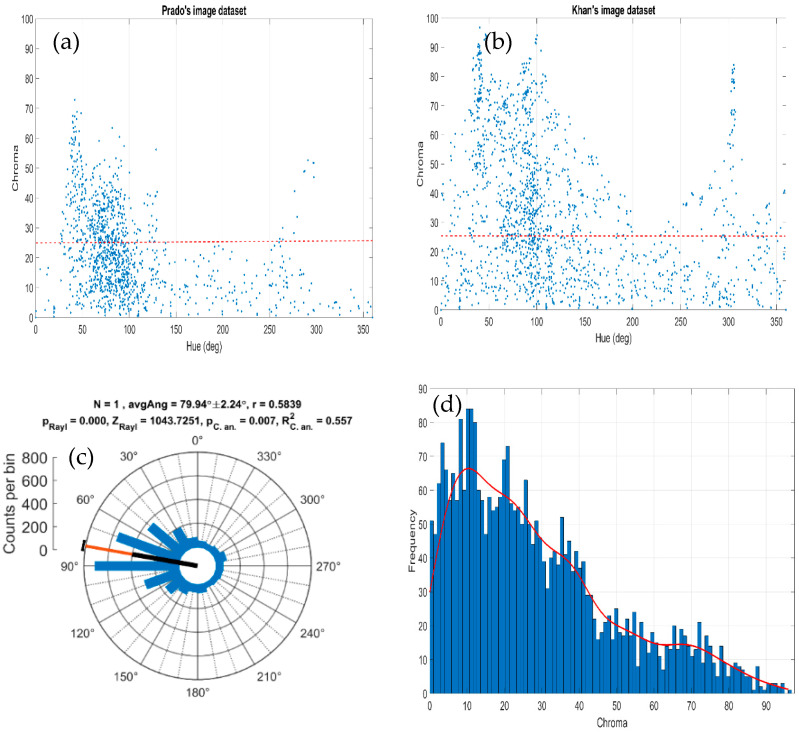
(**a**,**b**) Hue versus chroma colour components derived from the subjective relevant colours for all observers. (**c**) Polar histogram of the frequency distribution of the hue and (**d**) chroma colour components obtained for both sets of paintings and all observers.

Figure 8 shows all subjective relevant colours for all observers as a function of their corresponding hue and chroma values. For chroma values below 25 there was almost no hue preference in the subjective impression elucidated by each painting. Nevertheless, for chroma values above 25, we can see in the figure that the relevant colours selected by all observers correspond to some discrete set of hue components. The histograms in Figure 8 plot the distribution of directions in the hue plane and the chroma values for all observers. The results suggest that the hue values of the relevant colours cluster around some directions in colour space; there were different hue regions with higher density around 30°, 40°, 70°, 90°, 110°, and 130°, and another two relevant regions appeared around 150° and 290°. The histogram of chroma values shows that 70% of the total observations were below chroma values of 35, which is close to the 25 chroma value mentioned before.

Are those distributions of preferred directions in the hue plane connected somehow to some categorical hue components? To address this issue, we applied ICA to the whole of Khan’s image dataset. If we denote by *I^k^*(*x*,*y*) the pixel values in each painting image (*k* being the R, G, and B colour components), the ICA algorithm derives a representation of that image as a linear superposition of feature vectors *A_i_*:

(1) $$I^{k}\left(x,y\right)=\sum_{i=1}^{m}A_{i}^{k}\left(x,y\right)s_{i},$$

where *s_i_* denotes the coefficients for each image patch describing the independent sources, which are linearly combined into the observations *I^k^* through the basis function $A_{i}^{k}$. In matrix form, we can express this as

(2) I=As,

where *I* is the input colour data (i.e., the RGB values for each pixel), *s* is the statistically independent sources, and *A* is the mixing matrix. The goal of ICA is to infer *A* and solve for *s*:

(3) $$s=A^{-1}I=WI,$$

where the columns of *A* are usually called the basis functions and the rows of *W* are called the filters.

Following on from the above equations, we extracted the basis functions of the public Khan et al. [22] image dataset by using the FastICA Matlab package. A total of 273,000 patches (around 53 to 96 8 × 8 image patches per painting included in the dataset) were extracted from random locations from the collection of 4266 paintings in Khan’s set. The images were used as they were included in the original database, which means that no additional calibration and/or post-processing was used. They were pre-processed by approximative orthogonalization pre-whitening, and the FastICA algorithm was then used to extract basis functions that were as independent as possible. The basis functions, extracted as independent sources, were finally ordered by their energy levels and then were normalized.

**Figure 9:**
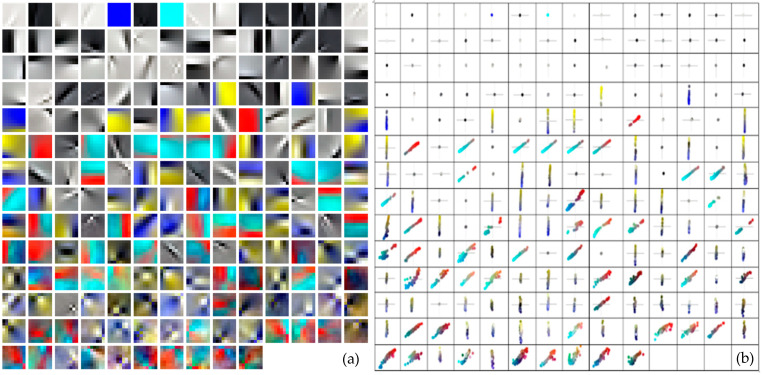
(**a**) Pseudo-colour representation of the spatio-chromatic structure of the activation for all 192 learned independent component analysis (ICA) basis functions. Each colour component corresponds to the R, G, and B pixel values for each 7 × 7 patch that was derived from individual basis functions. (**b**) Chromaticities of activation of each individual patch when projected onto a red-green and blue-yellow plane in the RGB colour space. The functions are in order of decreasing *L*^2^ norm from left to right and top to bottom.

Figure 9 shows the 192 learned ICA basis functions obtained and the chromaticities of every individual patch when projected onto a red-green (*RG*) and blue-yellow (*BY*) plane. Both colour planes were defined as

(4) $$RG=\left(\frac{1}{\sqrt{2}}\right)\left(R-G\right),$$

(5) $$BY=\left(\frac{1}{\sqrt{6}}\right)\left(R+G-2B\right),$$

where *R*, *G*, and *B* are the RGB colour values of every pixel in the images [34].

As expected from early studies [25,26,28], most of the obtained ICA bases show an oriented spatial distribution and can be divided into homogeneous patches, colour-opponent patches, and achromatic patches. Homogenous patches in Figure 9 can be either homogeneous chromatic or achromatic patches without any localized spatial structure; when they are plotted in the chromaticity diagram, colour coordinates cluster around the origin into a single point (see the first three top rows in the left and right plots in Figure 9). On the contrary, colour-opponent patches were characterized by a clear localized spatial structure, which means that pixel chromaticities of each patch were clustered along lines crossing the origin and dividing the chromaticities into two opponent quadrants (see examples in the lowermost rows in the Figure 9 plots). The fact that also no purely opponent chromatic directions (i.e., distributions of colours along the horizontal and vertical axes) appeared suggest that painting images elicited some colour-preferred distribution of colours along other cardinal directions. This behaviour agrees with previous studies that analysed the basis functions derived from natural scenes [33], showing that the distribution of colour preferences is not solely restricted to the red–green and yellow–blue colour-opponent chromatic axes.

**Figure 10:**
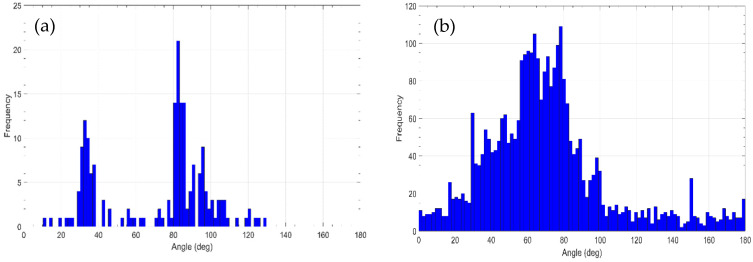
(**a**) Histogram of the distribution of chromatic directions associated to each ICA basis function using the Khan set of painting images. (**b**) Histogram of colour angles in the RG and BY colour plane obtained from the relevant colours and all observers used in the psychophysical experiment.

Figure 10a shows the histogram of preferred colour directions across directions in colour space as derived from the ICA. Each direction was determined by computing the first principal component of the distribution of 8 × 8 × 3 pixel values from each of the 192 ICA bases. The directions shown by the histogram are almost identical to the colour directions found for natural scenes by Kellner and Watchler [33], as shown in their Figure 7 in that paper. The main difference is the lobe we get around 35°, probably due to the more colourful images contained in Khan’s set in comparison with the natural images used in that work. To further analyse whether the relevant colours obtained in the psychophysical experiment could be colour instances derived from those preferred colour directions, we determined for each relevant colour its RG and BY colour components according to Equations (4) and (5) and computed the corresponding colour angle as

(6) $$\theta =\tan^{-1}\left(\frac{BY}{RG}\right).$$

Figure 10b shows the angle values obtained, suggesting a relatively good agreement with the preferred colour directions obtained with the ICA. This fact supports the hypothesis that observers selected the most relevant colour content of the paintings following an efficient coding.

## 4. Discussion and Conclusions

The results we found in this experiment illustrate how observers are able to pick the most relevant colours that describe the subjective impression gained by the observation of paintings. Because colour memorization can be more accurate for those colours described by a higher density of colour names [35], observers were instructed to directly select those pixels belonging to a relevant chromatic area and were not compelled to put names to all their choices. If “memory colour tended to be more characteristic of the dominant chromatic attribute of the objects”, it is plausible that observers would put in the table that memory to select their relevant colours [36]. It is the familiar, naturally occurring colours that influence the observers’ selections. The subjective relevant colours found could be considered as a colour categorical perception [37], that is, the discriminability of some colours over others is favoured according to the semantic content or subjective impression of the painting evoked in the observer. We found a small average number of 21 colours to describe the subjective impression of the colours that compose the colour palette of paintings. This is a very small number in comparison with the potentially thousands of colours that a human observer could discriminate [15]. Obviously, the number of discernible colours (as defined, for instance, by Linhares et al. (2008)) would not necessarily represent all those colours that an observer would have to use to describe image colours. In fact, the average number of objects per scene “that may be discriminated by their colour is about one-fifth the number of discriminable colours” [38]. On the contrary, that number of relevant colours is clearly above the classical 11 colour names used in colour naming approaches, which was already demonstrated by our early computational analysis [20], but is in agreement with other machine learning approaches that proposed around 32 colours for better object classification [19]. That relatively small number of relevant colours reflects somewhat the bottleneck of visual information processing in the visual pathway. Although the human visual system is able to process 109 bits/second at the retina level, this number dramatically decays to around 40 bits/second at higher visual areas [39].

The results suggest that the complex spatio-chromatic content of the images does not have to correspond to the selection of a higher number of relevant colours. Examples like *The Triumph of Death* by Pieter Bruegel the Elder (oil on panel, 1562–1563; painting number 17, fourth row, in Figure 1) elicited almost the same number of relevant colours as did other paintings apparently much more visually “simple”. This means that observers focused more on the representative colours of the painter’s palette, ignoring the spatio-chromatic complexity of the painting. On the contrary, simpler paintings (like some examples from Khan’s set in Figure 1) would a priori cause a more random observer response given the simplicity of the paintings and the apparent reduced number of objects, figures, and colours; this behaviour could originate from the presence of large colour areas in the painting (not really totally uniform), making it difficult to get similar RGB relevant values among observers. Besides this, the average number of subjective relevant colours agrees with that previously estimated computationally by Nieves et al. [20]. In that work, we used large colour differences to segment the colour gamut of a painting, and by imposing restrictions on the L* and chroma values for each pixel, we developed an algorithm to obtain images described by very few relevant colours. In the current work, the distribution of hue components in each painting particularly influences the final choice of the relevant colours and could be an a priori feature to determine the most representative colours describing the palette of paintings.

Additionally, our results show that observers were particularly keen at selecting remarkable hue and chroma colour components. We found that the hue values of all subjective relevant colours found in this study clustered around some directions in colour space, particularly along 30°, 40°, 70°, 90°, 110°, and 130°. Does this mean that new canonical colour dimensions should be added to the existing opponent ones? Unless we use cone excitations from painting images, we cannot conclusively say that. Rather, those directions may resemble eye tracking experiments that indicate how observers would perform longer fixations on yellow and brown colours and shorter fixations on orange, green, and black colours [40]. These results are also in good agreement with the distributions of opponency directions found by Kellner and Wachtler [33] using natural scenes. By applying ICA, these authors showed that chromatic selectivity is more continuously distributed in colour space. They found a distribution of chromatic preferences around 90° (i.e., light-blue and dark-yellow colours), 130° (i.e., orange and teal colours), 65–80°, and, to a lesser extent, around 10–30° (i.e., reddish colours) in the cone excitation space. Furthermore, the midget retinal ganglion cells have been proven as ideal hue detectors accurately encoding surface colours and edges [41]. Yet, somehow, our relevant colours mimicked the more efficient spatio-chromatic manifested by the human visual system when natural images are processed in the visual cortex.

In follow-up experiments, we will study whether the relevant colours actually lead to a higher correlation than just naïvely picking equally spaced colours in each painting. The number of uniformly selected colours that would be necessary to reach the same correlation found here between the subjectively coloured image and the original one would highlight to what extent the subjective colours give an efficient representation of the colours of each painting.

## Figures and Tables

**Table 1:** Statistical descriptors (average and standard deviation) derived from the two image datasets used. Row number 1 presents data from the Prado set, and row number 2 present the results from the Khan set.

Set	NDC	NRC	L*	a*	b*	Angle (°)	Ratio	Area	Self-Similarity	Complexity	Anisotropy	Birkhoff Metric	Volume
1	56,754	19	35.0	4.2	12.9	67	0.41	2011	0.685	2.5	8.47 × 10^−7^	0.283	142,216
SD		5	9.8	3.7	4.3	17	0.11	1431	0.085	0.6	3.03 × 10^−7^	0.051	59,450
2	77,125	21	56.3	2.9	14.3	74	0.49	7528	0.612	4.4	1.62 × 10^−6^	0.190	337,709
SD		7	16.1	8.5	12.2	26	0.15	6923	0.246	3.2	1.21 × 10^−6^	0.156	141,009

SD stands for the standard deviation, NDC for the number of discernible colours, and NRC for the number of relevant colours.

## Supplementary Materials

The following are available online at https://www.mdpi.com/article/10.3390/jimaging7040072/s1, Figure S1: Coverage percentage of the overlapping between the distribution of computational relevant colors and the subjective colors found in the experiment, Figure S2: the average number of relevant colors (NSC) found in the experiment against each aesthetic metric used (see Section 2.4) for (top figure) the Prado set and (bottom figure) the Khan set.

## References

[1] Graham D.J., Redies C. (2010). Statistical regularities in art: Relations with visual coding and perception. Vis. Res..

[2] Wallraven C., Fleming R., Cunningham D., Rigau J., Feixas M., Sbert M. (2009). Categorizing art: Comparing humans and computers. Comput. Graph..

[3] Mureika J.R., Dyer C.C., Cupchik G.C. (2005). Multifractal structure in nonrepresentational art. Phys. Rev. E.

[4] Yelizaveta M., Tat-Seng C., Irina A. Analysis and retrieval of paintings using artistic color concepts. Proceedings of the 2005 IEEE International Conference on Multimedia and Expo.

[5] Montagner C., Linhares J.M., Vilarigues M., Nascimento S.M. (2016). Statistics of colors in paintings and natural scenes. JOSA A.

[6] Nascimento S.M., Linhares J.M., Montagner C., João C.A., Amano K., Alfaro C., Bailão A. (2017). The colors of paintings and viewers’ preferences. Vis. Res.

[7] Romero J., Gómez-Robledo L., Nieves J. (2018). Computational color analysis of paintings for different artists of the XVI and XVII centuries. Color Res. Appl..

[8] Li C., Chen T. (2009). Aesthetic visual quality assessment of paintings. IEEE J. Sel. Top. Signal Process..

[9] Frey H., Honey C., König P. (2008). What’s color got to do with it? The influence of color on visual attention in different categories. J. Vis..

[10] Heckbert P. (1982). Color image quantization for frame buffer display. Siggraph Comput. Graph..

[11] Google Arts & Culture. https://artsandculture.google.com/.

[12] Colormind. http://colormind.io/.

[13] Simoncelli E.P., Olshausen B.A. (2001). Natural image statistics and neural representation. Annu. Rev. Neurosci..

[14] Graham D.J., Field D.J. (2007). Efficient neural coding of natural images. New Encycl. Neurosci..

[15] Linhares J.M.M., Pinto P.D., Nascimento S.M.C. (2008). The number of discernible colors in natural scenes. JOSA A.

[16] Masaoka K., Berns R.S., Fairchild M.D., Abed F.M. (2013). Number of discernible object colors is a conundrum. JOSA A.

[17] Witzel C., Gegenfurtner K.R. (2018). Color perception: Objects, constancy, and categories. Annu. Rev. Vis. Sci..

[18] Párraga C., Benavente R., Baldrich R., Vanrell M. (2009). Psychophysical measurements to model intercolor regions of color-naming space. J. Imaging Sci. Technol..

[19] Yu L., Zhang L., van de Weijer J., Khan F.S., Cheng Y., Parraga C.A. (2018). Beyond eleven color names for image understanding. Mach. Vis. Appl..

[20] Nieves J.L., Gomez-Robledo L., Chen Y., Romero J. (2020). Computing the relevant colors that describe the color palette of paintings. Appl. Opt..

[21] Prado Museum. https://www.museodelprado.es/en/the-collection.

[22] Khan F.S., Beigpour S., Van de Weijer J., Felsberg M. (2014). Painting-91: A large scale database for computational painting categorization. Mach. Vis. Appl..

[23] Psychtoolbox 3. http://psychtoolbox.org/.

[24] Eizo Color Edge CG277. https://eizo.es/producto/cg277-coloredge/.

[25] Nodine C., Mello-Thoms C., Krupinski E., Locher P. (2008). Visual interest in pictorial art during an aesthetic experience. Spat Vis..

[26] Van Geert E., Wagemans J. (2020). Order, complexity, and aesthetic appreciation. Psychol. Aesthet. Creat. Arts.

[27] Hugo GävertJarmo HurriJaakko SäreläAapo Hyvärinen, FastICA Matlab Package. http://research.ics.aalto.fi/ica/fastica/.

[28] Nieves J.L., Romero J. (2018). Heuristic analysis influence of saliency in the color diversity of natural images. Color Res. Appl..

[29] Barlow H.B. (1961). Possible principles underlying the transformation of sensory messages. Sens. Commun..

[30] Bell A.J., Sejnowski T.J. (1997). The “independent components” of natural scenes are edge filters. Vis. Res..

[31] Nadal J., Parga N. (1993). Information processing by a perceptron in an unsupervised learning task. Netw. Comput. Neural Syst..

[32] Wachtler T., Lee T., Sejnowski T.J. (2001). Chromatic structure of natural scenes. JOSA A.

[33] Kellner C.J., Wachtler T. (2013). A distributed code for color in natural scenes derived from center-surround filtered cone signals. Front. Psychol..

[34] Ruderman D.L., Cronin T.W., Chiao C. (1998). Statistics of cone responses to natural images: Implications for visual coding. JOSA A.

[35] Hasantash M., Afraz A. (2020). Richer color vocabulary is associated with better color memory but not color perception. Proc. Natl. Acad. Sci. USA.

[36] Bartleson C.J. (1960). Memory Colors of Familiar Objects*. J. Opt. Soc. Am..

[37] Hanley J.R. (2015). Color categorical perception. Encycl. Color Sci. Technol..

[38] Foster D.H. (2018). The Verriest Lecture: Color vision in an uncertain world. JOSA A.

[39] Zhaoping L. (2006). Theoretical understanding of the early visual processes by data compression and data selection. Netw. Comput. Neural Syst..

[40] Fontoura P., Menu M., Schaeffer J.M. (2021). Visual perception of natural colors in paintings: An eye-tracking study of Grünewald’s Resurrection. Color Res. Appl..

[41] Patterson S.S., Neitz M., Neitz J. (2019). Reconciling color vision models with midget ganglion cell receptive fields. Front. Neurosci..

