# Supplementary material for: Psychophysical Determination of the Relevant Colours That Describe the Colour Palette of Paintings
Source: J Imaging. 2021 Apr 14;7(4):72. doi: 10.3390/jimaging7040072 (PMC8321366; doi:10.3390/jimaging7040072)
Supplement: Supplementary file 1 [file jimaging-07-00072-s001.pdf]

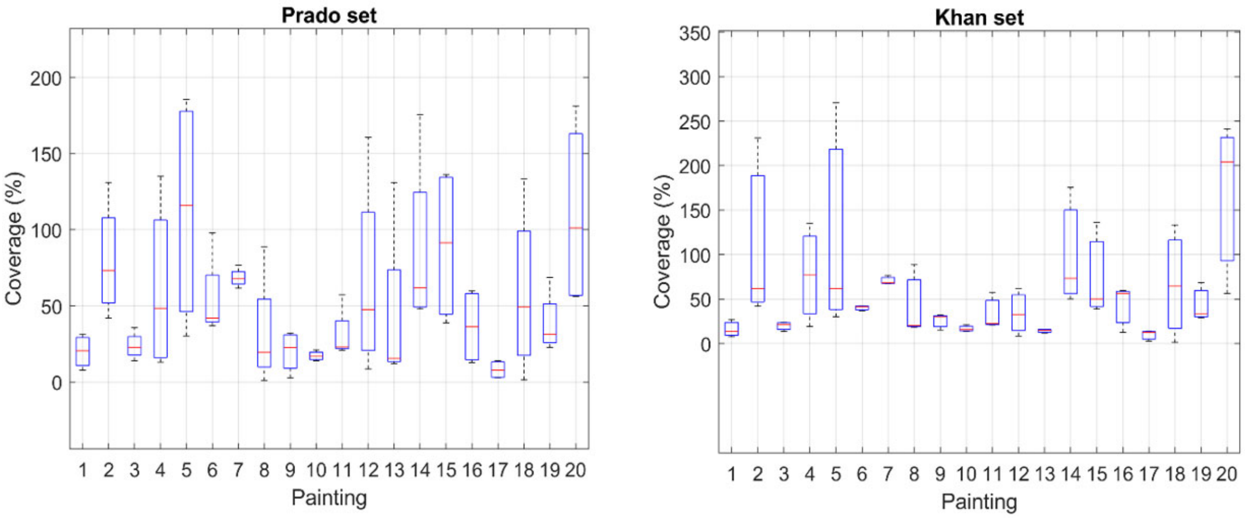

Figure S1. Coverage percentage of the overlapping between the distribution of computational relevant colors and the subjective colors found in the experiment (i.e. ratio comparing the intersection area between the subjective gamut and the computational gamut and the area of the subjective gamut).

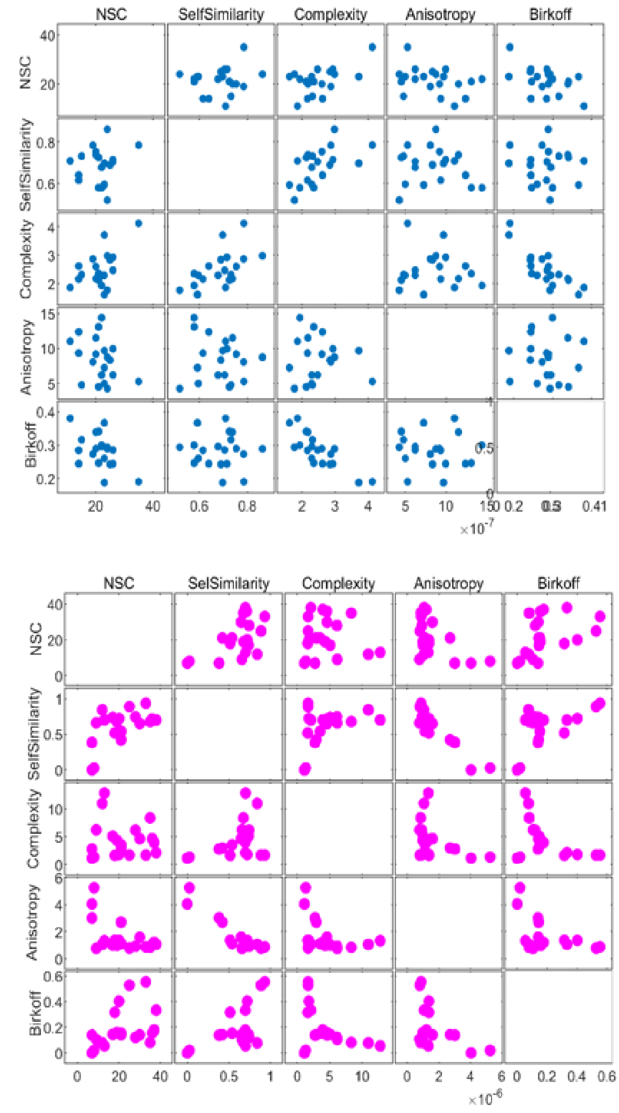

Figure S2. The plots below show the average number of relevant colors (NSC) found in the experiment against each aesthetic metric used (see Section 2.4) for (top figure) the Prado set and (bottom figure) the Khan set. .
